# A Sustainable and Flexible Carbon Paper-Based Multifunctional Human–Machine Interface (HMI) Sensor

**DOI:** 10.3390/polym17010098

**Published:** 2025-01-01

**Authors:** Muhammad Muqeet Rehman, Maryam Khan, Hafiz Mohammad Mutee ur Rehman, Muhammad Saqib, Shahzad Iqbal, Sang Seop Lim, Kun Hyun Park, Woo Young Kim

**Affiliations:** 1 Department of Electronic Engineering, Faculty of Applied Energy System, Jeju National University (JNU), Jeju City 63243, Republic of Korea; maryamkhan93@stu.jejunu.ac.kr (M.K.); saqibmuhammad@jejunu.ac.kr (M.S.); shahzadiqbal@stu.jejunu.ac.kr (S.I.); 2 Department of Mechanical Engineering, School of Mechanical and Manufacturing Engineering (SMME), National University of Science and Technology (NUST), Islamabad 44000, Pakistan; muhammad.mutee@smme.nust.edu.pk; 3 iTACT, Jeju City 63357, Republic of Korea; sseop@itact.co.kr (S.S.L.); 3313park@itact.co.kr (K.H.P.)

**Keywords:** flexible carbon paper, hydrophilic porous structure, sustainable electronic device, scalable fabrication, human–machine interface (HMI)

## Abstract

We have executed a cost-effective approach to produce a high-performance multifunctional human–machine interface (HMI) humidity sensor. The designed sensors were ecofriendly, flexible, and highly sensitive to variability in relative humidity (%RH) in the surroundings. In this study, we have introduced a humidity sensor by using carbon paper (as both a substrate and sensing material) and a silver (Ag) conductive ink pen. The carbon paper-based humidity sensor was developed by using a simple handwriting approach and the resulting devices exhibited excellent results including fast response/recovery times (12/24 s), a wide sensing range (30 to 85%), small hysteresis (1.1%), high stability (1 month), and repeatability. This high-performance humidity response could be attributed to the highly porous, hydrophilic, and permeable nature of carbon paper. Besides these features, the sensor offered high flexibility (100 bending cycles across different radii) and adaptability for uses like breath monitoring (through mouth and nose), proximity sensing (from multiple distances ranging from 1 to 10 cm), and depicting Morse code. This research work is a significant step forward in humidity sensing technology and the sustainable future of electronic devices by using a cost-effective, fast, and simple fabrication technique.

## 1. Introduction

Humidity sensors play an important role in our daily life and across a variety of industries such as food production, healthcare services, agriculture, automotive sector, electronic industry, heating, ventilation, air conditioning (HVAC) systems, textile manufacturing, printing industry, energy sector, museums, construction fields, and aerospace technology [1,2,3,4,5,6,7,8,9,10,11,12,13]. Humidity sensors help to maintain moisture levels to ensure safety, efficiency, and quality control by preventing food spoilage, safeguarding medications, improving crop yields, ensuring comfort levels, and safeguarding costly machinery. Industrial applications of humidity sensors include preventing static electricity damage, preserving historical artifacts, maintaining structural stability, and supporting avionic systems. In healthcare settings, noninvasive humidity sensors assist with diagnostics while the paper manufacturing industry relies heavily on proper moisture regulation. Studies are looking into cutting-edge materials such as polymers, metal-organic frameworks (MOFs), two-dimensional (2D) materials, and nanomaterials to improve sensitivity, low humidity detection capabilities, and the integration of IoT (Internet of Things) [14,15,16,17]. The focus is on miniaturizing humidity sensors with more flexibility to utilize them for wearable and self-powered electronics for human–machine interface (HMI) applications.

Paper is a famous choice among materials for humidity sensors due to its favorable attributes including cost-effectiveness, sustainability, fast absorption/desorption of water molecules (due to cellulose fibers), high flexibility, and porous structure [18]. Paper-based humidity sensors can be easily produced using methods like inkjet printing or screen printing as well as the “pencil on paper” technique. New improvements in fabrication techniques and material modification have successfully addressed challenges associated with paper-based humidity sensors such as conductivity and uneven surface textures. When cellulose was mixed with other materials like graphene oxide (GO) and carbon nanotubes (CNTs), it improved the performance of sensors due to its internal structure and stability as a natural polymer. Several research teams have developed humidity sensors on paper by applying different types of materials onto the paper surface. Zhao et al. [19] reported a humidity sensor that utilized a combination of TiO_2_ and silver nanoparticles (Ag NPs) to improve sensitivity and hydrophilic properties. This humidity sensor proved to be a viable option for the application of safeguarding documents and artworks. Solak et al. [20] developed a humidity sensor system using paper to monitor exhaled breath and differentiate between the breathing behaviors of smokers and non-smokers. Their sensor was tested on individuals with pneumonia or chronic obstructive pulmonary (COP) disease that involved 101 participants (with 41 being patients). Xue et al. [21] fabricated a paper-based humidity sensor that was designed to be highly sensitive using an interdigitated electrode (IDE) capacitor resonator. The resonator was fine-tuned through high-frequency structure simulator (HFSS) simulation software and improved with GO films for increased sensitivity. Copper (Cu)-coated paper was also used in the design of this sensor that achieved a change of 6.25 MHz per %Relative Humidity (%RH) within the humidity range of 60 to 90%RH. Eryanto et al. [22] developed a self-powered humidity sensor using Cu and aluminum (Al) electrodes along with a magnesium chloride-carbon nanotube (MgCl_2_-CNT) composite on paper with the ability to detect humidity levels ranging from 11 to 97%RH. This paper-based sensor boosted response times and outputs at 1.07 V with a power generation capability of 2 μW. Guan et al. [23] developed a cellulose paper-based environment-friendly humidity sensor that was modified with chemicals to enhance its sensitivity and reduce response time to just 25 s (s), thus making it ideal for overseeing gadgets (like optical switches) and measuring skin moisture levels. Bhattacharjee et al. [24] introduced a device for lung function monitoring point-of-care-testing (LFM-POCT) that included a paper-based humidity sensor and had the ability of real-time monitoring. This POCT device also included a micro heater to measure the frequency and peak flow rate of air for the detection of chronic obstructive pulmonary diseases (COPDs). Duan et al. [25] developed a humidity sensor made from printing paper and flexible conductive tape that resulted in excellent sensitivity for multifunctional applications. Lin et al. [26] developed a highly cost-effective paper-based humidity sensor through inkjet printing technology that utilized original black ink for electrodes and sodium chloride (NaCl) for the sensing layer application. This sensor was designed to monitor humidity levels in respiration, skincare, and gas detection (in bird farming environments) applications at a cost of less than 1 EUR.

From the above discussion, we can easily deduce that the paper-based humidity sensors typically use paper as a substrate only and try to enhance its conductivity through modification with conductive materials like GO, CNTs, TiO_2_/Ag NPs, PEDOT: PSS, and PANI for improved functionality. These paper-based humidity sensors are often developed using advanced methods like inkjet or screen printing. In this study, we have tackled almost all the mentioned challenges related to paper-based humidity sensors by integrating carbon paper (instead of normal paper) into our sensor design. Firstly, we employed carbon paper as both the substrate and active layer in our humidity sensor design. Secondly, we refrained from altering the carbon paper with any other material/toxic chemical due to its naturally higher conductivity (~10^2^ S/m) as compared to typical paper (~10^−4^ S/m). This approach allowed us not only to steer clear of conductive or harmful materials but also to streamline the manufacturing process significantly. Lastly, we applied a handwriting method for the development of IDEs using Ag ink on carbon paper, thus eliminating the need for sophisticated fabrication printing techniques. This innovative yet simple fabrication design leveraged the qualities of carbon paper to develop a humidity sensor that delivered fast response/recovery times while maintaining high stability over a broad humidity range. A multitude of uses are possible for this sensor including breath monitoring (through mouth and nose), proximity sensing, and depicting Morse code interpretation. The outstanding durable nature and hydrophilic properties of carbon paper with dense porosity made it a perfect fit for producing affordable humidity sensors that could also be used in wearable and IoT gadgets. In general, our simple fabrication technique combined with the excellent electronic/mechanical performance of the carbon paper-based sensor represented progress in practical humidity sensing technologies for HMI applications.

## 2. Experimental Section

### 2.1. Materials

Carbon paper (Star Starred, Goyang, Republic of Korea) was used as the substrate and active layer of the developed humidity sensor in its pristine form without any further processing. Highly conductive (resistance 0.05–0.2 Ω/sq/mil) and non-toxic Ag ink roller ball pens (Circuit Plus, Seoul, Republic of Korea) were used for patterning IDEs. Ag epoxy (4Science Co., Ltd., Seongnam, Republic of Korea) was used to make strong contact with Cu wires with the IDEs for electrical characterization. Transparent and flexible silicone (Si) adhesive tape (4Science Co., Ltd., Seongnam, Republic of Korea) was used to enhance flexibility and mechanical robustness. A commercially available reference humidity sensor of HTU21D was used for the calibration of our sensor.

### 2.2. Characterizations and Measurements

The surface morphological analysis of carbon paper was examined by using a field emission scanning electron microscope (FESEM) (Phenom Pharos G2, ThermoFisher Scientific, Waltham, MA, USA) while Fourier transform infrared spectroscopy (FTIR) was used for its chemical analysis (BRUKER TENSOR 27, Ettlingen, Germany). These morphological and material characterization methods provided useful information about the composition and properties of the carbon paper, at both quantitative and qualitative levels. The FTIR spectra were captured across a range of 450–4500 cm^−1^ using potassium bromide (KBr) pellets to ensure precise data collection accuracy was maintained. We tested the flexibility of our developed humidity sensor by bending it at different angles repeatedly using different radii across a pen surface. This testing configuration provided simple conditions for assessing the durability and flexibility of our humidity sensor. Additionally, we also tested the mechanical robustness of our sensor by twisting it for multiple cycles by holding its two ends. The electrical characteristics of the developed humidity sensor were measured by placing it in sealed jars of super-saturated solutions carrying different salts (calcium chloride, potassium carbonate, sodium bromide, sodium chloride, and copper chloride) resulting in 30, 40, 55, 62, 73, and 85%RH, respectively, along with the commercially available reference humidity sensor (HTU21D). The humidity sensor’s electrical response to varying %RH levels was measured by using the KEYSIGHT U1733C LCR meter to record impedance and capacitance changes.

### 2.3. Device Fabrication

**Figure 1:**
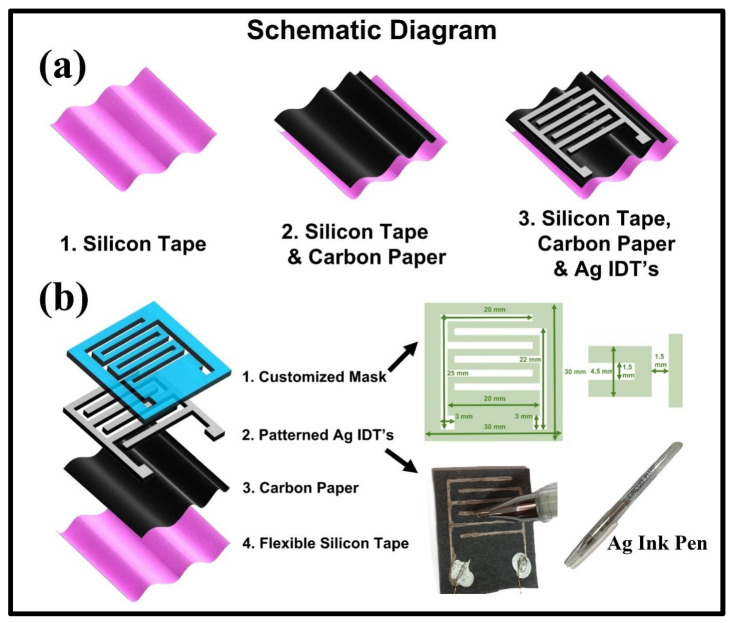
(**a**) Schematic diagram of device fabrication. (**b**) Layered structure of a carbon paper-based humidity sensor, a detailed labeled diagram of the customized mask along with the optical images of a conductive Ag ink pen, and our developed sensor.

We fabricated three different samples of our carbon paper-based humidity sensor to check its repeatability by cutting the carbon paper into the same sizes. The schematic diagram of our facile device fabrication technique is shown in Figure 1a. Multiple samples of carbon paper with the dimensions of 3 cm × 3 cm were made for patterning Ag IDEs on their surface. The cut pieces of carbon paper samples were attached to a flexible and transparent Si adhesive tape to further strengthen the mechanical robustness of the carbon paper substrate. A customized steel mask was placed on the surface of each sample whose detailed dimensions are presented in Figure 1b. Highly conductive IDEs were patterned on the surface of each carbon paper sample by using a commercially available Ag ink pen as shown by the optical images presented in Figure 1b. Two thin copper wires were attached at the contacts of Ag IDEs by using Ag epoxy for electrical characterization of the developed sensors. All three developed sensors were then placed on the hot plate at 60 °C for 1 h (in an open lab environment) to dry Ag epoxy, which resulted in stronger contact. In this way, carbon paper served dual purposes of substrate and functional thin film for sensing humidity. A notable point here is that the highly conductive Ag IDEs were patterned by hand on the surface of carbon paper with the help of a commercially available low-cost conductive ink pen and customized mask. A single Ag ink pen could make IDE patterns over 60 to 200 m (depending on the paper surface being used), implying that thousands of samples can be made by using a single Ag ink pen that only costs ~USD 4.5. Additionally, the cost of a single sheet of A4 size carbon paper (enough for making dozens of humidity sensors) was only ~USD 0.8, making the overall cost of our humidity sensor extremely low at less than ~USD 0.1, whereas the price of a commercially available humidity sensor like HTU21D can be in the range of ~USD 5 to USD 15 (depending on the supplier). It should be noted that the whole process of patterning Ag IDEs took a total time of less than 2 min hence, proving the fact that the fabrication process was fast and facile. Furthermore, no chemical or hazardous material was used in the entire fabrication process of the potato peel-based humidity sensor hence, making it a biocompatible and user-friendly humidity sensor. The above-described fabrication process of our humidity sensors clearly shows that our proposed technique was highly cost-effective, less time-consuming, and extremely simple.

## 3. Results and Discussions

### 3.1. Material Characterizations

**Figure 2:**
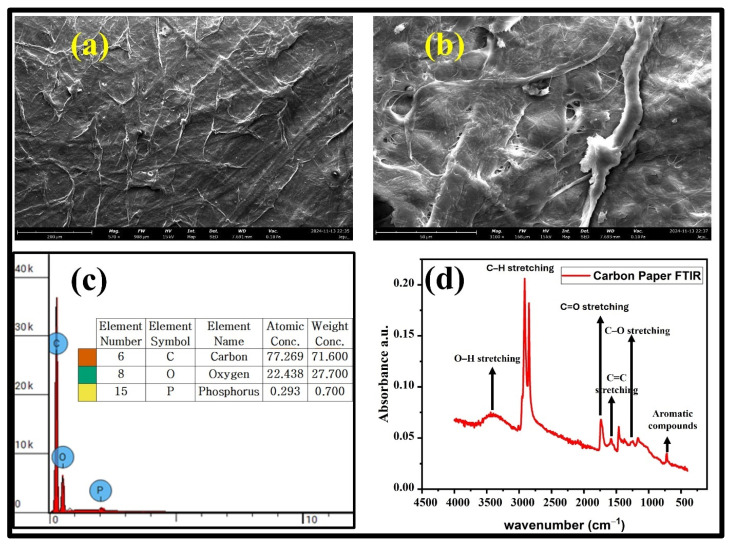
Morphological analysis of carbon paper by using FESEM at (**a**) 200 µm and (**b**) 50 µm. (**c**) Elemental composition of carbon paper with wt% of each element present in it. (**d**) FTIR spectrum of carbon paper depicting the presence of important functional groups for sensing humidity.

SEM characterization of carbon paper at 200 µm and 50 µm resolutions highlighted its potential as an effective humidity-sensing layer as shown in Figure 2a,b. The porous and interconnected fibrous network of carbon paper significantly increased the surface area (enhancing moisture adsorption) and sensitivity to humidity changes. Pronounced surface roughness promoted wettability and efficient moisture absorption, while the hydrophilicity of this fibrous structure ensured strong interaction with environmental moisture content. Microchannels between fibers enabled rapid capillary action and uniform moisture retention, while the compact structure of carbon paper ensured good electrical conductivity for effective signal generation. Additionally, the robust and flexible framework of carbon paper (visible even at higher magnifications), supported mechanical durability for flexible devices. These multi-scale features made carbon paper ideal for humidity sensors with high performance and long-term stability.

The elemental composition of carbon paper was also examined, and it turned out to be 71.6 wt% carbon (C), 27.7 wt% oxygen (O), and 0.7 wt% phosphorus (P), as shown in Figure 2c. These elements play a critical role in the performance of carbon paper as a humidity-sensing material. Carbon (C) provides a robust fibrous network, enhanced conductivity (compared to conventional paper), and a high surface area, enabling efficient charge transfer and water molecule adsorption. Oxygen (O) introduces hydrophilic groups (hydroxyl, carbonyl, carboxyl), boosting water affinity, rapid adsorption/desorption, and impedance-based sensitivity. The presence of all these functional groups was confirmed by obtaining the FTIR spectra of carbon paper as shown in Figure 2d. Traces of phosphorus (P) contribute through surface functionalization, forming phosphate groups that improve ionic conductivity, hydrogen bonding, and humidity responsiveness while enhancing chemical stability. Together, these elements synergistically balance the conductivity, sensitivity, and durability of a humidity sensor thus, making carbon paper a reliable material for advanced humidity sensor applications.

The FTIR spectrum of carbon paper was analyzed from 450 to 4500 cm^−1^ and it revealed a rich array of functional groups that signify its effectiveness as a humidity-sensing material. Key peaks include 3442 cm^−1^ (O–H stretching). This peak indicated the presence of hydrophilic hydroxyl groups that are important for water molecule adsorption via hydrogen bonding. Prominent peaks at 2923 cm^−1^ and 2852 cm^−1^ were attributed to C–H stretching vibrations of alkyl groups, with 2923 cm^−1^ corresponding to asymmetric stretching of CH_2_ groups and 2852 cm^−1^ to symmetric stretching, respectively. These peaks suggested the presence of aliphatic hydrocarbons or residual organic compounds, likely from binders, processing agents, or surface treatments during fabrication. Another important peak was observed at 1743 cm^−1^ (C=O stretching), reflecting carbonyl groups that helped to enhance sensor sensitivity through hydrogen bond formation. The peak at 1242 cm^−1^ (C–O stretching) represented phenolic and carboxylic groups that contribute to the hydrophilicity and electrical responsiveness of the humidity sensor. The peaks at 1576 cm^−1^ and 720 cm^−1^ confirmed the presence of aromatic structures that ensured structural stability, while the peak at 1159 cm^−1^ confirmed ether linkages that aid in the humidity interaction. Together, these hydrophilic and hydrophobic features balance rapid water interaction with stable conductivity, enabling efficient and reliable humidity sensing. This functional versatility makes carbon paper a robust material for advanced sensor applications.

### 3.2. Electrical Characterization

**Figure 3:**
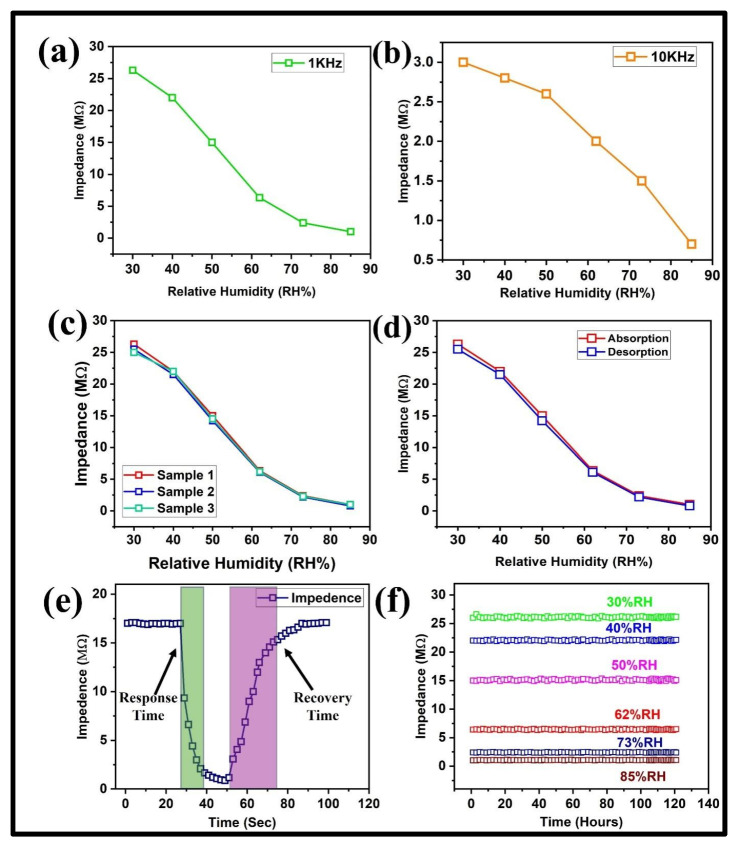
Electrical characterization of a carbon paper-based humidity sensor. (**a**) Impedance response at 1 kHz. (**b**) Impedance response at 10 kHz. (**c**) Repeatability of multiple humidity sensors. (**d**) Small hysteresis between the adsorption and desorption of a humidity sensor. (**e**) Response and recovery time obtained from the dynamic response of a humidity sensor. (**f**) Stable impedance response of a humidity sensor at multiple %RH.

The functionality of the humidity sensor made from carbon paper was tested by performing its multiple impedance measurements to determine its sensitivity levels and stability over time. We tested the impedance response of our sensor at two different frequencies of 1 kHz and 10 kHz, as shown in Figure 3a and Figure 3b, respectively; however, the change in impedance at a lower frequency (1 kHz) was more notable (27 to 1 MΩ) than its response (3 to 0.7 MΩ) at a high frequency (10 kHz) in the range of 30 to 85%RH. The lower sensitivity at higher frequencies was possibly due to the reduced ion flow. This change in impedance shown by our carbon paper-based humidity sensor was mainly due to the ability of carbon paper to absorb water vapors’ porous/fibrous structure. The concentration of water molecules sticking with the carbon paper surface kept increasing and occupied its pores with an increase in %RH. This whole process helped to boost the conductivity, leading to a decrease in the overall impedance of the carbon paper-based humidity sensor. Although our humidity sensor showed variation in its impedance value at both lower (1 kHz) and higher frequency (10 kHz), we selected the lower frequency of 1 kHz for further characterizations to enhance the detection of conduction impacts by moisture more efficiently. We developed three different humidity sensors by following the same fabrication technique and characterized all the samples by following the same method. The obtained results showed a highly consistent behavior across the %RH range as shown in Figure 3c. This overlapping impedance response of multiple sensors confirmed the high repeatability of our carbon paper-based humidity sensor that can be linked to the steady absorption of moisture and consistent electrical pathways, in each sample tested.

The release of water molecules is equally important for a humidity sensor as the adsorption of water molecules on its surface; therefore, we plotted the response of both adsorption and desorption of a carbon paper-based humidity sensor. An extremely low hysteresis value of only 1.2% was obtained as shown in Figure 3d. Low hysteresis value ensured precise, accurate, and reliable humidity sensing. The hysteresis value further helped to explore the difference between adsorption and desorption processes in relation to impedance and %RH. The high surface energy and capillary action in the microstructure of carbon fibers enabled moisture exchange, thus reducing any retention effects that might have caused small hysteresis. The response/recovery time of sensors is the most important feature that must be determined for their successful implementation in different applications. We determined the response and recovery time of our carbon paper-based humidity sensor by plotting its dynamic response. Response time was determined as the time taken for the signal to rise from 10% to 90% of its maximum value, while recovery time was determined as the time for the signal to drop from 90% to 10%. The obtained results showed a response and recovery time of 12 s and 24 s, as shown in Figure 3e, respectively. The interconnected pore structure of carbon paper helped in the speedy diffusion of water molecules. A slower recovery time (24 s) compared to the response time (12 s) in a carbon paper-based humidity sensor suggested faster diffusion of humidity but slower release of water vapors, likely due to strong water adsorption by hydrophilic groups. We determined the stability of our carbon paper-based humidity sensor over a long period of 1 month at different humidity levels (30%, 40% 50%, 62%, 73%, and 85%) and the obtained results showed high stability over time as shown in Figure 3f. The durability, stable microstructures, and sturdy nature of carbon paper could withstand changes in the environment to ensure moisture interaction and electrical efficiency. It can be easily deduced from the obtained electrical characterization results of carbon paper-based humidity sensors that it exhibited high sensitivity and stability due to the unique characteristics of carbon paper, making it a good choice for various suitable applications.

### 3.3. Conduction Mechanism

The conduction mechanism of a carbon paper-based humidity sensor is a somewhat complex process that could involve both electronic and ionic conduction. The carbon paper’s conductivity, hydrophilic surface with functional groups, and porous structure enable efficient water adsorption and the formation of a conductive water layer. The structure and functional groups of carbon paper played a vital role in sensing humidity changes effectively. The elements of O (27.3 wt%) and C (71.6 wt%) were mainly responsible for hydroxyl (–OH) and carbonyl (C=O) functional groups that assist in water absorption via hydrogen bonding that could initiate ionic conduction with rising humidity levels. Moreover, the presence of P (0.7 wt%) in a relatively smaller amount boosted ionic conduction, thereby enhancing the sensitivity of carbon paper-based humidity sensors further. The interaction of carbon paper with moisture significantly alters the sensor’s electrical properties like impedance, thus allowing it to accurately detect and monitor changes in humidity. The porous and rough surface of carbon paper speeds up moisture transfer leading to fast response and recovery times. Carbon paper stands out among other conventional paper types for its higher conductivity and ability to handle moisture well. Additionally, the flexibility of carbon paper enables its use in cutting-edge sensing technologies like RF and IoT systems.

### 3.4. Mechanical Characterization

**Figure 4:**
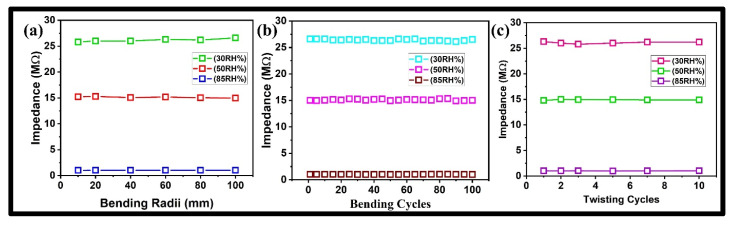
Mechanical robustness of a carbon paper-based humidity sensor (**a**) Stability against multiple bending radii at different %RH. (**b**) Stability against multiple bending cycles at different %RH. (**c**) Stability against multiple twisting cycles at different %RH.

Carbon paper is naturally resilient against mechanical stress and fatigue. We tested the mechanical robustness of a carbon paper-based humidity sensor by measuring its impedance output at different %RH levels after twisting and bending it at different radii to scale its performance in real-world applications like wearable electronics where it could face unintended deformations. Our experimental findings showed that the carbon paper-based humidity sensor upheld its impedance values after being subjected to physical strain. We folded our sensor across the pens with different radii (100 to 10 mm) and checked its stability at multiple %RH (30, 50, and 85). The obtained results clearly showed that the impedance values of the carbon paper-based-humidity sensor were the same after bending as they were originally when the sensor was not exposed to bending at different radii as shown in Figure 4a. Furthermore, we tested the stability of the carbon paper-based humidity sensor for 100 bending cycles at multiple %RH (30, 50, and 85), and the obtained results were highly stable as shown in Figure 4b. This consistent performance can be linked to the flexible nature of carbon paper structure known for its mechanical strength and adaptability. The carbon layers enhanced its durability by offering support that safeguards the pathways from any harm when bent repeatedly. Therefore, the moisture-sensitive qualities of the carbon paper persist unaltered even after multiple usage cycles. These results of mechanical characterization indicate that the conductive network of the carbon paper was not affected by bending. The fibrous network of carbon paper is naturally flexible to endure bending without damage or disruption to its pathways. Additionally, the existence of functional groups like hydroxyl (–OH) and carbonyl (C=O) present in the carbon paper does not notably change the sensors’ physical characteristics when bent or flexed mechanically. The relationship between water molecules and these functional groups remains unchanged during these deformations. These results have clearly shown that the carbon paper-based humidity sensor was highly durable and could easily withstand fatigue effectively. Furthermore, we exposed carbon paper-based humidity sensors to multiple twisting cycles by holding both ends with tweezers. The sensor was twisted 10 times, and its impedance response was determined at multiple %RH (35, 50, and 85). The obtained results exhibited that no considerable change in the impedance of the carbon paper-based humidity sensor occurred as shown in Figure 4c. The findings revealed that the impedance levels consistently maintained stability throughout the twisting trials making a strong case for the mechanical robustness of the sensor against strain. The reasons behind this stability can be attributed to the naturally occurring fibrous network of carbon paper that induces both flexibility and the ability to resist any deformations due to twisting. Carbon fibers can withstand shear forces well, enabling the sensor to maintain its properties even under torsional stress. The test results for the carbon paper-based humidity sensor show that it remains stable and reliable when bent or twisted due to its flexible structure with hydrophilic properties that help maintain its electrical functions during deformation.

### 3.5. Applications

**Figure 5:**
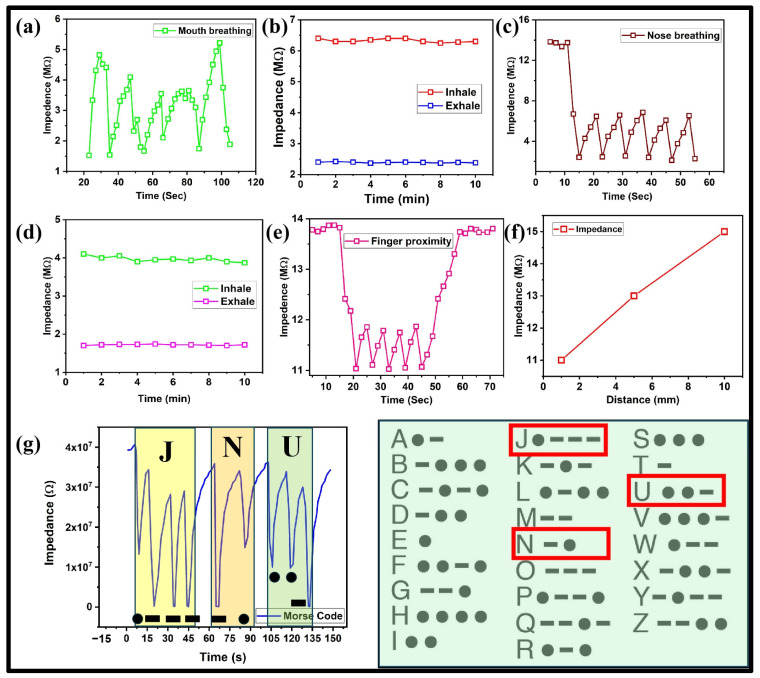
Multifunctional applications of carbon paper-based humidity sensor (**a**) Dynamic response of mouth breathing. (**b**) Stability of maximum/minimum impedance values during mouth breathing. (**c**) Dynamic response of nose breathing. (**d**) Stability of maximum/minimum impedance values during nose breathing. (**e**) Dynamic response of proximity sensing. (**f**) Impedance response of proximity sensing at different distance values. (**g**) Successful depiction of Morse Code and its standard table.

We tested the performance of a carbon paper-based humidity sensor in real-world scenarios because of its suitable sensitivity to %RH and flexibility. The sensor was utilized to monitor breathing cycles of exhaling and inhaling. Our sensor proved to be highly sensitive in that it could distinguish between breathing through the mouth and nose. The impedance values and their stability for 10 min during exhaling and inhaling through mouth and nose are shown systematically in Figure 5a–d. The obtained results clearly showed that a carbon paper-based humidity sensor is highly suitable for the application of health monitoring due to its biocompatibility and high sensitivity. Distinct patterns indicated during inhalation and exhalation align with variations in the moisture levels of the inhaled/exhaled air through the nose/mouth. Monitoring breath patterns could reveal important information related to sleep disorders (like sleep apnea), nasal blockages, respiratory infections, and abnormalities linked to nasal air passage in the healthcare field. The sensor could efficiently detect variations in impedance caused by breath humidity while keeping it close to the mouth/nose in an open lab environment to offer an invasive way for immediate monitoring. The fibrous and porous structure of carbon paper efficiently absorbs moisture in exhaled/inhaled breath, enabling swift and precise real-time monitoring of moisture content. The stability of the sensor was maintained due to the durability of carbon paper, making it a viable option. The carbon paper-based humidity sensor demonstrated excellent mechanical robustness, enduring 100 bending cycles across various radii without degradation in performance. Its high flexibility ensured that it could be easily integrated into wearable devices, making it highly suitable for real-world applications such as health monitoring, wearable HMIs, and proximity sensing.

The carbon paper-based humidity sensor was also tested for the finger proximity test that monitored its impedance changes as the finger was brought closer to and moved away from the active sensor area. The obtained results showed a dynamic response that clearly indicated that the impedance of the sensor decreased when the finger was brought close to the sensor and again increased when the finger was moved away from the sensor as shown in Figure 5e. Additionally, we measured the exact impedance value of our sensor by keeping our finger away at different distances (1 to 10 mm) that resulted in an impedance change (11 to 15 MΩ) as shown in Figure 5f. Carbon paper’s rapid humidity response, conductivity, and hydrophilic properties enable touchless finger movement tracking through moisture absorption from the skin. The impedance of the sensor kept increasing with the increasing distance of the finger. These test results showed how well the sensor could gauge distance by detecting impedance variations, which can be applied in proximity sensing scenarios, like switches and security systems, for homes and other smart applications. The finger proximity test could play an important role in applications involving human–machine interaction (HMI). This feature could enable control systems, user interfaces, and advanced HMI devices to detect finger presence and motion through humidity changes, thus enhancing accessibility.

The carbon paper-based device effectively showed Morse code depiction by sensing finger movements that matched dots and dashes. Longer peaks demonstrated dashes while shorter peaks demonstrated dots. We successfully depicted the right set of dots and dashes (in Morse code) for the three letters of JNU as shown in Figure 5g. This experimental result shows the ability of carbon paper-based humidity sensors to encode and send data effectively using Morse code through proximity detection for purposes like aiding communication for people with disabilities or enabling other similar communication setups.

## 4. Conclusions

A carbon paper-based humidity sensor was designed in this study that exhibited the highly favorable characteristics that made it a strong candidate for use in multifunctional applications. The sensor used the advantage of carbon paper’s intrinsic characteristics including its porous fibrous structure, its hydrophilic/hydrophobic functional groups, and the biocompatibility to give highly sensitive, stable, and reproducible responses to variations in relative humidity (%RH). The method of fabrication was rather facile and did not require much effort or sophisticated equipment as it involved the use of a handwriting technique with a conductive silver (Ag) ink pen and a customized steel mask. The humidity sensor also exhibited excellent electrical characteristics in that its impedance altered from 27 MΩ to 1 MΩ within a wide %RH range and with a low hysteresis of ~1.2%. The carbon paper-based humidity sensor exhibited a fast response/recovery time (12/24 s) with high stability of over 1 month to prove its reliability. Carbon paper is a flexible material, so we tested its mechanical characterizations as well by subjecting it to foldability and twisting tests. The obtained results were highly encouraging and paved the way for using our sensor in a diverse range of human–machine interface (HMI) applications. These HMI applications included breathing monitoring, proximity sensing, and Morse code depiction. The results obtained in this study support the concept of applying carbon paper-based humidity sensors in different areas of environmental control, the medical industry, and smart technologies, which present a low-cost, efficient, and effective solution for the next generation of sensors.

## Data Availability

Data are contained within the article.
